# Predisposition to *Ascaris lumbricoides* reinfection in the Geshiyaro project in southern Ethiopia

**DOI:** 10.1093/trstmh/trag075

**Published:** 2026-07-06

**Authors:** Olivia Simmonds, Rosie Maddren, Benjamin Collyer, Birhan Abtew, Ewnetu Firdawek Liyew, Melkie Chernet, Getachew Tollera, Geremew Tasew, Esayas Knife, Mahlet Belachew, Mesay Hailu, Yasin Awol, Roy M Anderson

**Affiliations:** Department of Infectious Disease Epidemiology, School of Public Health, Faculty of Medicine, Imperial College London, 90 Wood Lane, London W12 0BZ, United Kingdom; Department of Infectious Disease Epidemiology, School of Public Health, Faculty of Medicine, Imperial College London, 90 Wood Lane, London W12 0BZ, United Kingdom; Department of Infectious Disease Epidemiology, School of Public Health, Faculty of Medicine, Imperial College London, 90 Wood Lane, London W12 0BZ, United Kingdom; Department of Infectious Disease Epidemiology, School of Public Health, Faculty of Medicine, Imperial College London, 90 Wood Lane, London W12 0BZ, United Kingdom; Bacterial, Parasitic and Zoonotic Disease Research Directorate, Ethiopian Public Health Institute, Swaziland Street, 2PWJ P8C, Addis Ababa, Ethiopia; Bacterial, Parasitic and Zoonotic Disease Research Directorate, Ethiopian Public Health Institute, Swaziland Street, 2PWJ P8C, Addis Ababa, Ethiopia; Bacterial, Parasitic and Zoonotic Disease Research Directorate, Ethiopian Public Health Institute, Swaziland Street, 2PWJ P8C, Addis Ababa, Ethiopia; Bacterial, Parasitic and Zoonotic Disease Research Directorate, Ethiopian Public Health Institute, Swaziland Street, 2PWJ P8C, Addis Ababa, Ethiopia; Bacterial, Parasitic and Zoonotic Disease Research Directorate, Ethiopian Public Health Institute, Swaziland Street, 2PWJ P8C, Addis Ababa, Ethiopia; Bacterial, Parasitic and Zoonotic Disease Research Directorate, Ethiopian Public Health Institute, Swaziland Street, 2PWJ P8C, Addis Ababa, Ethiopia; Bacterial, Parasitic and Zoonotic Disease Research Directorate, Ethiopian Public Health Institute, Swaziland Street, 2PWJ P8C, Addis Ababa, Ethiopia; Bacterial, Parasitic and Zoonotic Disease Research Directorate, Ethiopian Public Health Institute, Swaziland Street, 2PWJ P8C, Addis Ababa, Ethiopia; Department of Infectious Disease Epidemiology, School of Public Health, Faculty of Medicine, Imperial College London, 90 Wood Lane, London W12 0BZ, United Kingdom

**Keywords:** *Ascaris lumbricoides*, Ethiopia, longitudinal studies, mass drug administration, reinfection, soil-transmitted helminths

## Abstract

**Objectives:**

Predisposition to reinfection is characterized by certain individuals repeatedly acquiring infection at higher rates than others in the population despite treatment. As endemic regions approach low prevalence levels, such individuals will act as reservoirs of infection and contribute to sustaining transmission cycles in communities. This study investigated evidence for individual-level predisposition to *Ascaris lumbricoides* infections using longitudinal data from the Geshiyaro project in southern Ethiopia.

**Methods:**

Longitudinal parasitological data collected over 7 years were analysed. Kendall’s tau (τ) correlation coefficient was used to assess temporal persistence of infection between successive survey rounds, and Kendall’s coefficient of concordance (W) was used to evaluate long-term stability in individuals’ relative infection risk over many years of survey rounds.

**Results:**

Positive correlations in infection status between survey rounds were observed (τ = 0.10–0.30, *P* < .001). Moderate concordance in infection ranking across the study period was also detected (W = 0.43, 95% CI: 0.42–0.45; *P* < .001), indicating persistent individual-level predisposition over many years of treatment and reinfection.

**Conclusion:**

These findings demonstrate sustained heterogeneity in infection risk within endemic communities. As transmission declines, identifying individuals predisposed to reinfection may help inform targeted or complementary ‘test-and-treat’ strategies to support interruption of *A. lumbricoides* transmission.

## Introduction

Soil-transmitted helminths (STH) are a group of intestinal parasites that are primarily transmitted through direct contact with, or ingestion of, soil contaminated with infective eggs or larvae. There are four predominant species, namely, *Ascaris lumbricoides, Trichuris trichiura, Anclyostoma duodenale*, and *Necator americanus*. STH infections are widespread in tropical and subtropical regions, affecting an estimated 1.5 billion people worldwide, of which 79 million individuals are estimated to live in endemic areas of Ethiopia.^1^ These infections disproportionally affect socioeconomically disadvantaged communities, where limited access to sanitation and healthcare exacerbates their spread. While STH infections are rarely fatal, they are associated with severe morbidity, where severity is correlated with the intensity of infection or worm burden. Associated morbidity includes anaemia, malnutrition, impaired cognitive development, abdominal pain, and acute intestinal obstruction.^2^

The WHO classifies STH as infections targeted for elimination as a public health problem. This goal is defined as reducing the prevalence of any moderate or heavy STH infection to <2%, as determined by the Kato–Katz diagnostic test. A true prevalence of 2% is thought to be the level at which transmission interruption (R_0_ < 1) may occur; in low-prevalence settings, more sensitive diagnostics such as PCR are recommended to accurately measure true prevalence.^3^ To achieve this target, the WHO 2030 roadmap for STH control identifies preventive chemotherapy for preschool-aged children (pre-SAC), school-age children (SAC), and women of child-bearing age as a core strategic intervention.^4^ Progress towards achieving this goal is reliant upon the WHO’s prophylactic strategy of administering albendazole annually or biannually to these at-risk groups. Repeated treatments are necessary because helminth infections do not trigger strong acquired immunity, which implies that reinfection post-treatment is common.^5,6^

Since the 1980s, longitudinal studies have consistently shown that some individuals in the population are predisposed to STH reinfection.^7–12^ One study in India revealed significant correlations between worm burdens before and after treatment, showing that individuals who were heavily infected initially tended to reacquire heavy worm burdens over an 11-month reinfection period. Analyses in this endemic area of West Bengal showed that *A. lumbricoides* infections were highly aggregated, with most worms concentrated in a relatively small fraction of the population who were predisposed to reinfection.^10^

The reasons for predisposition remain unclear but are thought to reflect a combination of behavioural, environmental, and genetic factors.^10–12^ Behaviours that increase the risk of infection acquisition, such as poor hygiene or ingestion of contaminated soil, may differ from those that facilitate transmission to others, such as open defecation and environmental contamination. Predisposed individuals may therefore contribute to sustaining transmission cycles by repeatedly reacquiring infection and reintroducing infectious material into the local environment.

The WHO guidelines currently focus on STH transmission elimination through mass drug administration (MDA).^4^ Over the past decade, Ethiopia has made substantial progress towards STH control through multiple rounds of MDA in endemic areas and has succeeded in decreasing the prevalence of STH infection.^1,13^ However, if drug donations from the pharmaceutical industry decline, mass prophylactic treatment of individuals irrespective of their infection status may no longer be the most efficient or cost-effective strategy as uninfected individuals will receive unnecessary treatment.^14^ Modifying treatment strategies to target communities and/or individuals that are predisposed to reinfection could become beneficial if antihelminth drug donations to low- and middle-income countries decline.^14^ Such an approach would have cost implications, as it would require a shift towards ‘test and treat’ strategies.

This paper evaluates the evidence for individual-level predisposition to *A. lumbricoides* infection following prolonged community-wide treatment, using data from participants in the Geshiyaro project in southern Ethiopia. This analysis draws on the largest and longest longitudinal STH study to date, with up to 7 years of follow-up of individual infection and treatment patterns.

## Materials and methods

### Study site and design

The data used in this analysis originate from the sentinel site surveys collected as part of the Geshiyaro project. The study has three intervention arms. Arm 1 and Arm 2 are located in the Wolayita Zone in Ethiopia’s South Ethiopia Regional State, and Arm 3 sites are positioned in the Oromia and Sidama regions bordering Wolayita (Figure 1). There are 22 districts that comprise Arms 1 and 2 (increased from 15 at baseline due to the redefinition of district boundaries) and three districts in Arm 3. Arm 1 interventions involve community-wide MDA (cMDA), improved water sanitation and hygiene (WaSH) infrastructure, which is above the existing government WaSH programme, and behavioural change communication. Arm 2 involves cMDA and the existing national ‘one WaSH’ programme’. Arm 3 is a control group and involves school-based MDA (sMDA) and the existing national ‘One WaSH National Program (OWNP)’. The full design of the project has been previously described.^15^

**Figure 1:**
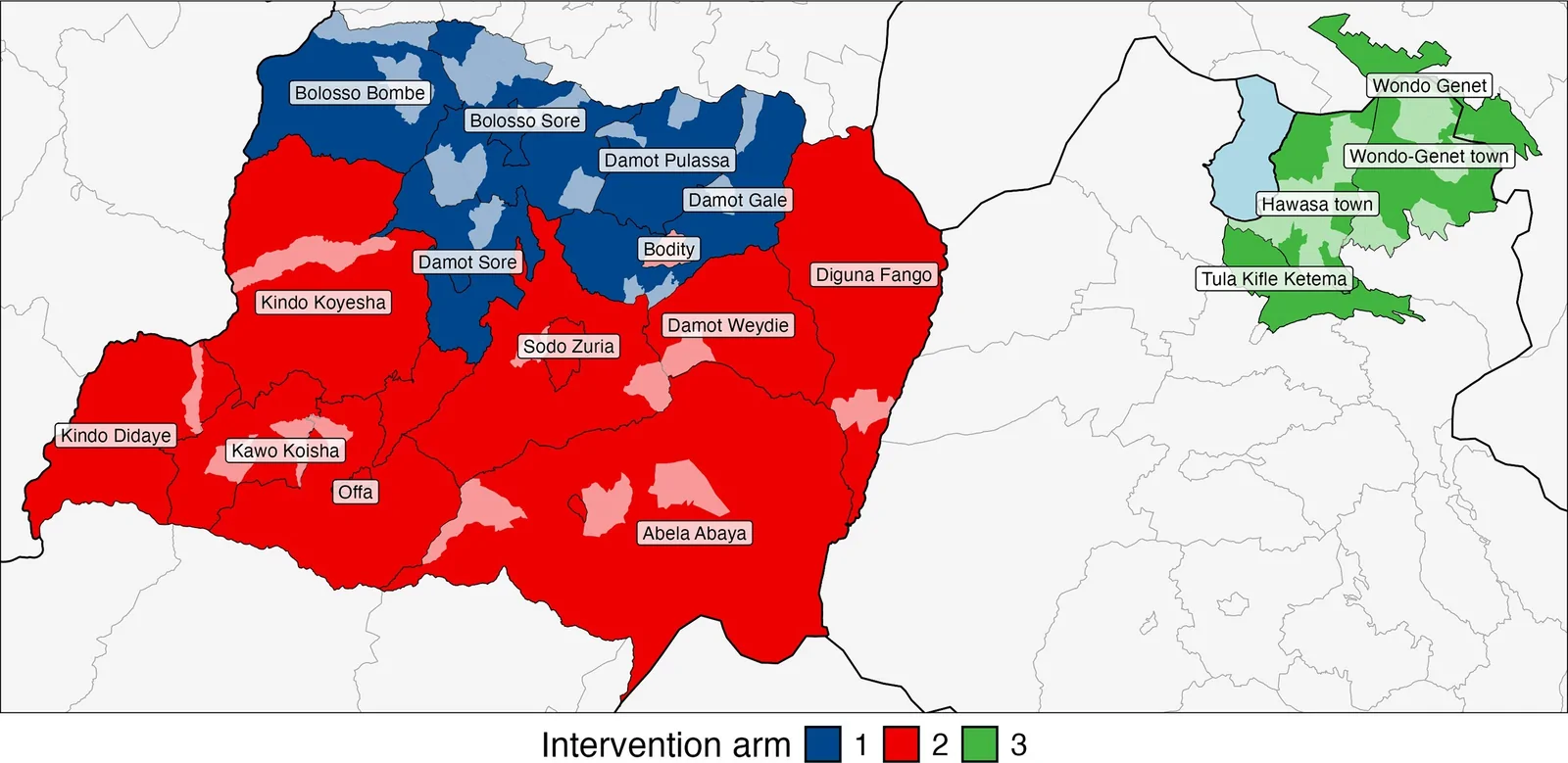
Location of the Geshiyaro project sites. Wolayita is divided into Arm 1 (blue) and Arm 2 (red). Arm 3 (green) sites are located in Oromia and Sidama. Woreda names are shown, and sentinel site locations are highlighted in pale shading. Lake Hawasa is coloured in light blue.

### Sentinel site surveys

The selection of longitudinal sentinel sites was based on baseline mapping, which stratified communities by low, moderate, and high STH prevalence, the methods of which have been previously described.^16^ Sites were selected at random from each co-endemicity category, resulting in 45 sentinel site communities across the three intervention arms. In each site 150 individuals were enrolled and followed longitudinally across survey rounds. If loss to follow-up occurred, individuals were replaced with participants of the same age and sex category to maintain the target sample size. The cohort was stratified by age (pre-SAC, 1–4 years; SAC, 5–14 years; 15–20 years; 21–35 years; **≥**36 years) and sex.

Sentinel site surveys began in January 2019 in four sentinel sites in the pilot district, Bolosso Sore, in Arm 1. In the remaining 26 sentinel sites in Arms 1 and 2, sentinel site surveys began in February 2020. Sentinel site surveys commenced in May 2021 for sites in Arm 3 (Supplementary Tables 1–3).

At each sentinel site survey, consenting participants provided two stool samples over two consecutive days. Samples were analysed using duplicate Kato–Katz slides to assess the presence of *A. lumbricoides* eggs.^16^ Infection intensity was quantified as the mean egg count from duplicate slides. This sampling procedure was repeated at each survey round, allowing longitudinal assessment of infection status within individuals over time. A detailed methodology for sample collection has been previously described.^16–18^ As *A. lumbricoides* was the dominant species across all study arms, analyses in this paper are restricted to *A. lumbricoides*.

### Mass drug administration

MDA with albendazole commenced in 2019 in the pilot district Bolosso Sore. Remaining woredas in Arm 1 and Arm 2 received the first round of treatment 1 year later in 2020. Albendazole was intended to be administered annually to all districts of the Wolayita zone; however, the delivery method was increased to biannual in 2022 to account for delayed MDA rounds during the COVID-19 pandemic. Treatment was offered to all individuals regardless of infection status. Arm 3 received school-based deworming through the OWNP in accordance with the WHO guidelines for STH and schistosomiasis control.

### Project timeline

The timing of monitoring activities and MDA interventions is summarized in Supplementary Tables 1–3. In Arms 1 and 2, aside from the baseline and first sentinel site survey, all surveys were preceded by cMDA. Because cMDA is expected to clear existing infections, infections detected at follow-up surveys reflect the acquisition of a new infection, rather than unresolved, persistent infections. Consequently, observed infections are interpreted as repeated reacquisition events and the analyses presented here assess the persistence of reinfection within the study population.

### Data analysis

Data analysis was performed in RStudio (R Version 4.4.2). In analyses, participants were stratified by age group, defined as pre-SAC (<5 years), SAC (5–14 years), and adults (**≥**15 years). The analyses were restricted to *A. lumbricoides* infections because it is the most prevalent STH species. Between each pair of consecutive sentinel site surveys, individuals were categorized as never infected, became infected, remained infected, and those that cleared their infection.

Kendall’s tau (τ) correlation coefficients were estimated using the correlation package in R to assess the temporal stability of individual infection status across sequential sentinel site surveys. This analysis evaluated the concordance between parasitological results obtained in two consecutive survey years, testing whether infection status tended to change in the same direction over time. Kendall’s tau ranges from −1 (complete discordance) to +1 (perfect concordance), with values near zero indicating no consistent association.

To assess longer-term stability in individuals’ relative infection rankings across all survey rounds, Kendall’s coefficient of concordance (W) was calculated using the DescTools package in R. Kendall’s W quantifies the degree of agreement among repeated measurements of infection status by assessing whether individuals maintain a consistent relative ranking over time. Values of W range from 0 (no concordance) to 1 (perfect concordance), with higher values indicating greater stability in relative infection burden across survey rounds. A bootstrap approach with 2000 resamples was used to generate 95% CIs (boot package). Kendall’s tau and Kendall’s W were used because they are both non-parametric rank-based statistics, and suitable for binary infection data, not requiring normally distributed data.

Differences in Kendall’s W between study arms/woredas/sentinel sites were assessed using 2000 bootstrap samples. Individuals were resampled with replacement within each group, W was calculated for each group, and pairwise differences (ΔW) were derived. Differences were considered significant if the 95% CI of ΔW did not include zero.

The association between the strength of individual-level predisposition (Kendall’s W) and the proportion of infected individuals per woreda/sentinel site/age group was assessed using Spearman’s rank correlation. For all tests reported herein, statistical significance was set at the 5% level (*P* ≤ .05).

## Results

A total of 9487 unique individuals had *A. lumbricoides* infection data recorded over the 7 years of the Geshiyaro project, with 3461 individuals in Arm 1, 3238 in Arm 2, and 2788 in Arm 3. The average prevalence in each intervention arm across all project years is summarized in Supplementary Fig. 1.

### Changes in infection status between adjacent sentinel site surveys

For each pair of consecutive sentinel site surveys, participants were categorized into one of four infection profiles: remained infected, became infected, lost infection, or those that were never infected. Changes in infection status between surveys are shown in Figure 2. Across all survey rounds, participants in Arm 3 had a significantly higher probability of remaining infected between consecutive survey rounds compared with those in Arms 1 and 2 (χ² = 3402–3748, *P* < .001).

**Figure 2:**
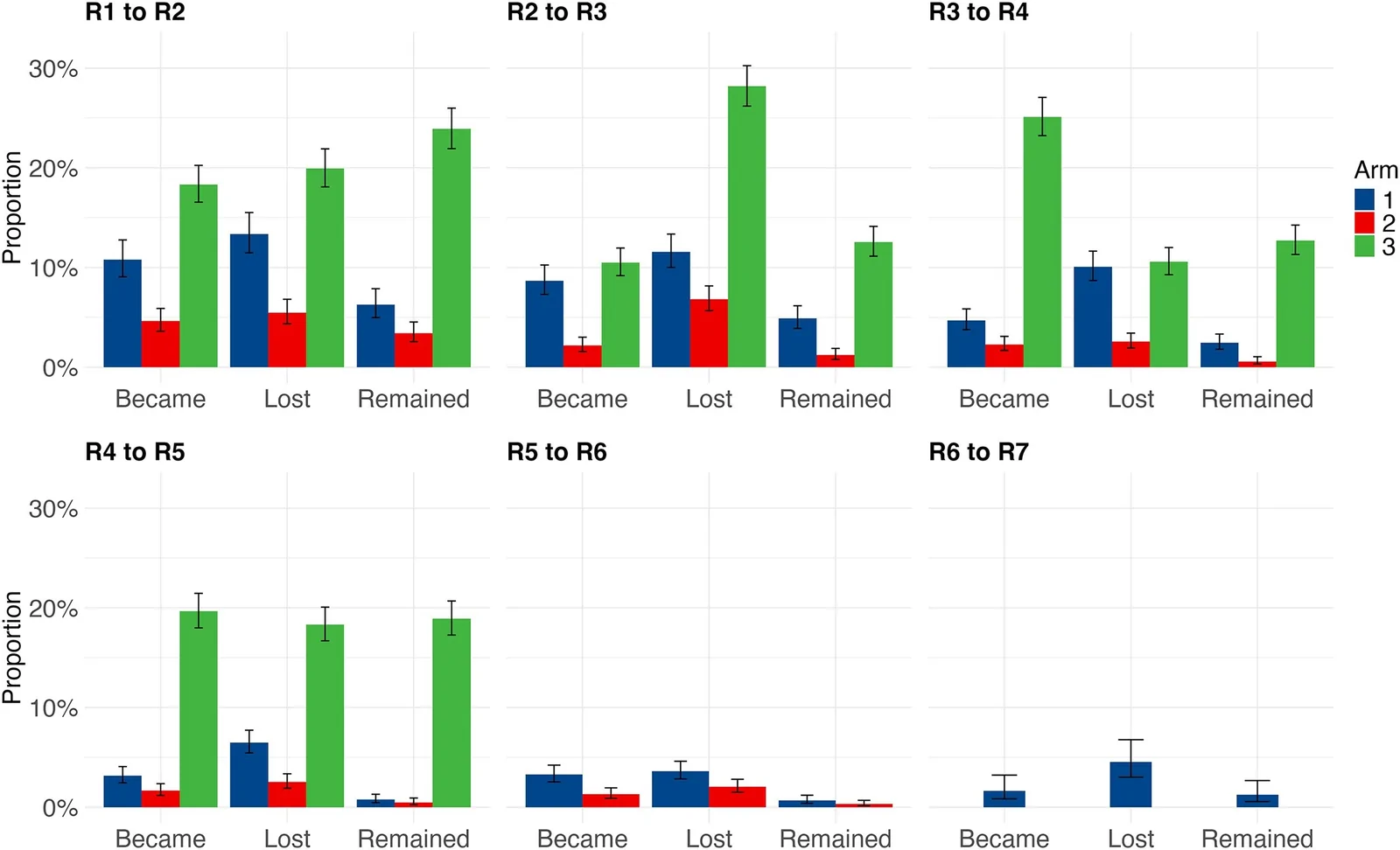
Changes in *A. lumbricoides* infection status between consecutive sentinel site surveys for individuals that became infected (became), lost infection (lost), and remained infected (remained), stratified by intervention arm.

### Temporal stability of infection status

Pairwise correlations in infection status were positive across all survey rounds (τ = 0.05–0.35, *P* < .05). The strongest associations were observed between temporally adjacent surveys and progressively weaker correlations between surveys separated by longer intervals. This pattern was consistent across all intervention arms (Figure 3A–C).

**Figure 3:**
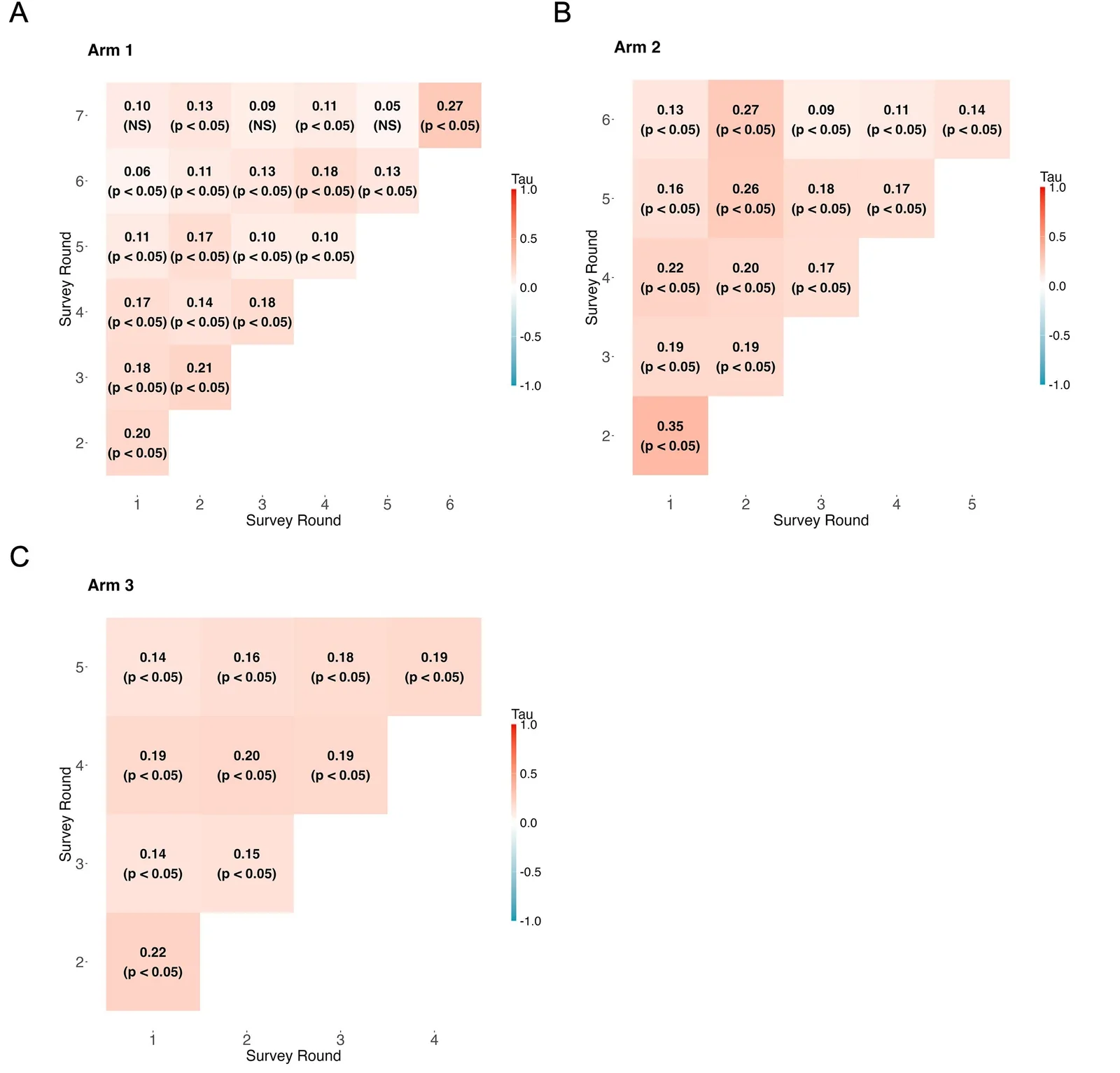
Heatmap of Kendall’s tau correlations between survey rounds for (A) Arm 1, (B) Arm 2, and (C) Arm 3. Each cell represents the correlation between the survey round on the x-axis and the survey round on the y-axis. Values inside cells denote Kendall’s τ, with statistical significance shown beneath (NS = not significant). The colour scale ranges from 0 (blue) to 1 (red).

### Individual-level predisposition to infection

Among all individuals in the longitudinal dataset, a moderate consistency in infection ranking across surveys was seen (Kendall’s W = 0.44, 95% CI: 0.43–0.45, *P* < .001). Evidence for predisposition was consistently observed in all arms (Figure 4): Arm 1 (W = 0.37, 95% CI: 0.35–0.39, *P* < .001), Arm 2 (W = 0.39, 95% CI: 0.36–0.42, *P* < .001), and Arm 3 (W = 0.39, 95% CI: 0.37–0.40, *P* < .001). Pairwise differences in W were small and included zero (Arm 1 vs Arm 2 ΔW = –0.019 to 0.020; Arm 2 vs Arm 3 ΔW = –0.043 to 0.012; Arm 2 vs Arm 3 ΔW = –0.030 to 0.039), indicating no significant difference in the degree of predisposition between arms.

**Figure 4:**
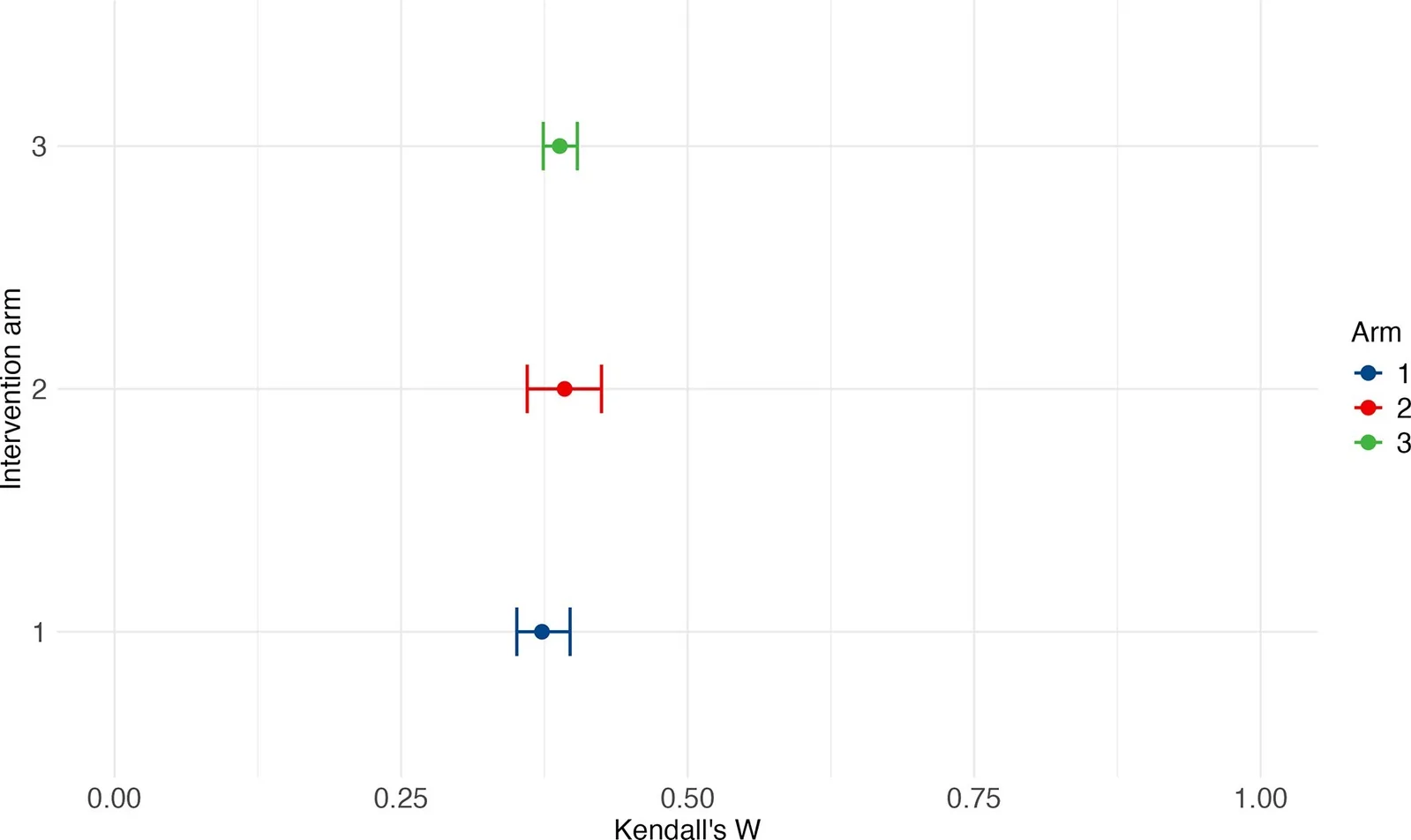
Kendall’s coefficient of concordance (Kendall’s W) across study arms. Points show the estimated Kendall’s W for each arm, with horizontal error bars indicating the corresponding confidence intervals calculated using 2 000 bootstrap resamples.

### District-level predisposition analysis

The strength of individual-level predisposition varied across woredas within each intervention arm (Figure 5). W values differed significantly between woredas in Arm 1 and Arm 2, whereas woredas in Arm 3 showed no significant differences in strength of W (Supplementary Tables 4–6). In Arm 1, there was no evidence of an association between the proportion of infected individuals and Kendall’s W (Spearman’s ρ = 0.50, *P* = .45). In Arm 2, Kendall’s W increased with the proportion of infected individuals (Spearman’s ρ = 0.86, *P* = .02). In Arm 3, the small number of woredas limited statistical power (Spearman’s ρ = -0.50, *P* = 1.00).

**Figure 5:**
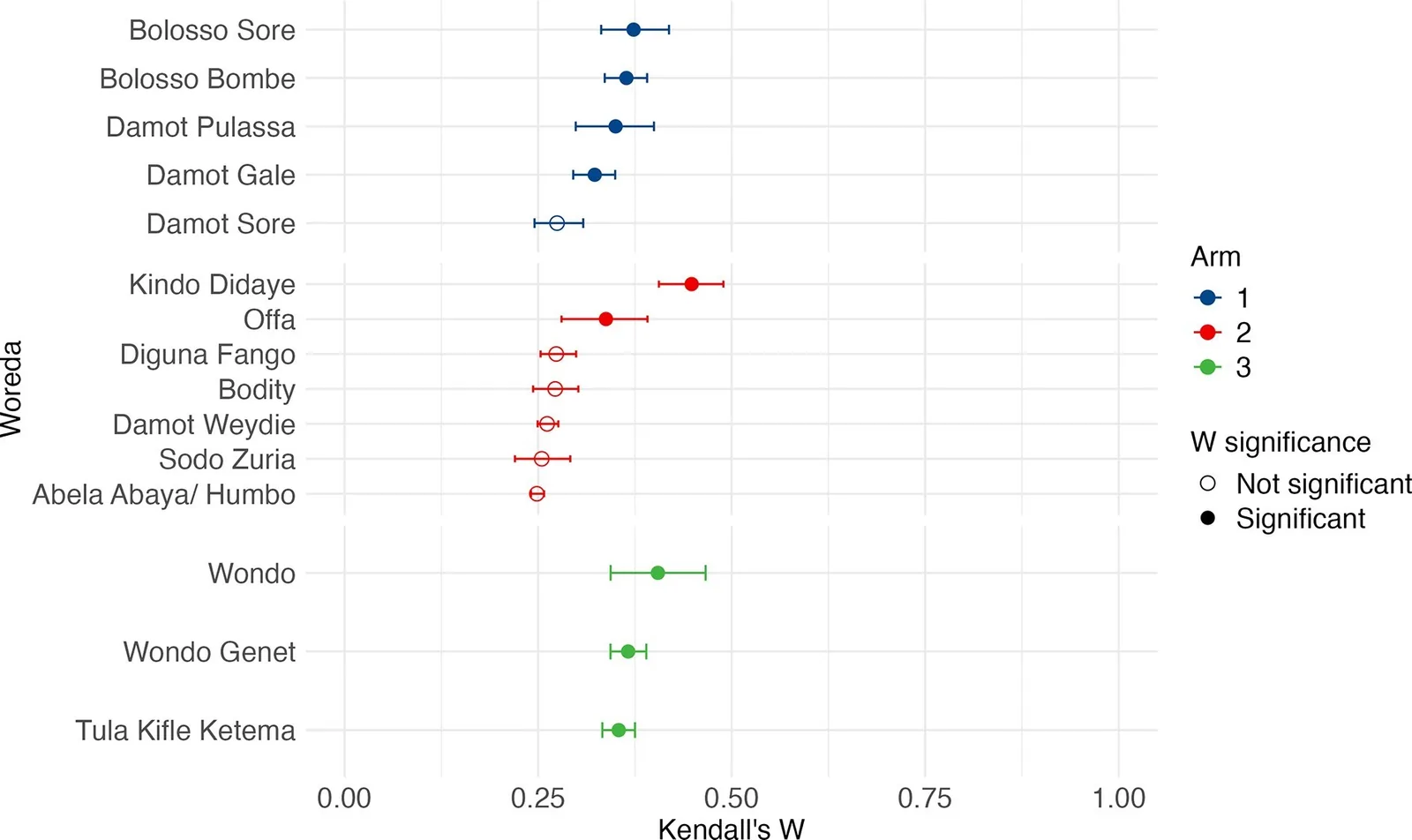
Strength of predisposition (Kendall’s W) by woreda in each intervention arm with 95% confidence intervals calculated using 2 000 bootstrap resamples. Filled points indicate W is significantly greater than zero; hollow points indicate W is not significantly different from zero.

### Kebele-level predisposition analysis

The strength of individual-level predisposition varied across sentinel sites within each intervention arm (Figure 6). Infection prevalence also varied between sites, raising the possibility that differences in W values reflect variation in the number of infected individuals rather than heterogeneity in predisposition. Across all sentinel sites combined, higher infection prevalence was associated with higher W values (Spearman’s ρ = 0.38, *P* = .01). However, this relationship was not observed when analyses were stratified by intervention arm.

**Figure 6:**
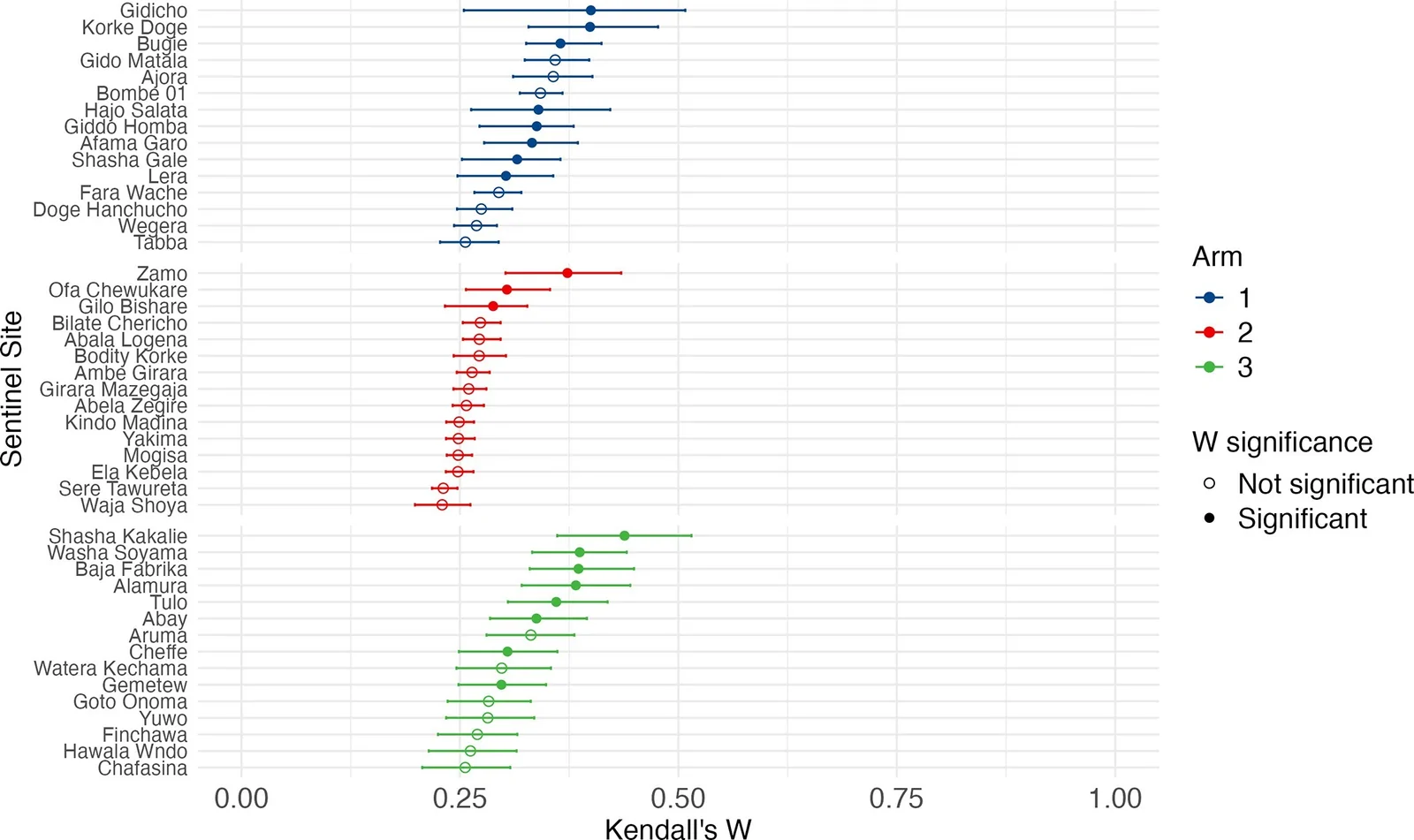
Strength of predisposition (W) stratified by kebele (sentinel site) in each intervention arm with 95% confidence intervals calculated using 2 000 bootstrap resamples.

### Age-stratified predisposition analysis

Moderate concordance in infection status was observed across all age groups (Figure 7). Kendall’s W was highest among individuals aged 15–20 years (W = 0.49, 95% CI: 0.45–0.52, *P* < .001) and lowest in people aged ≥36 years (W = 0.38, 95% CI: 0.35–0.41, *P* < .001). Pairwise comparisons of W between age groups indicated that differences were small and largely non-significant, except for a modest reduction in W in the oldest age group relative to younger groups (Supplementary Table 7). Infection prevalence showed a weak, non-significant correlation with W across age groups (Spearman’s ρ = −0.40, *P* = .52), and comparable sample sizes suggest that differences in W were not driven by unequal group sizes.

**Figure 7:**
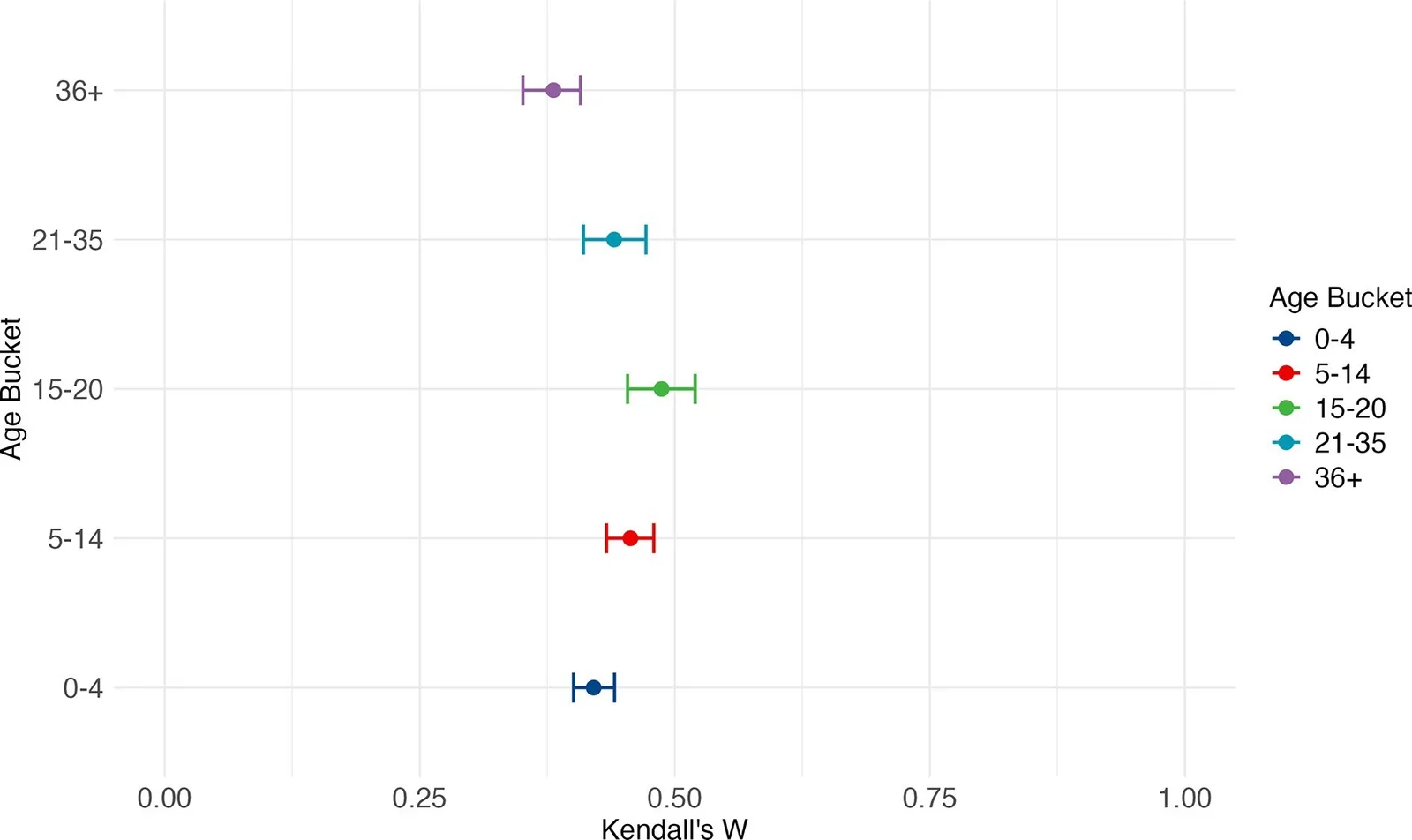
Strength of predisposition (W) within each age group with 95% confidence intervals as calculated using 2 000 bootstrap resamples.

### Sex-stratified predisposition analysis

Both males (W = 0.43, 95% CI: 0.41–0.45, *P* < .001) and females (W = 0.44, 95% CI: 0.42–0.46, *P* < .001) showed moderate evidence of predisposition (Supplementary Fig. 2). Furthermore, there was no significant difference between the W values and, hence, strength of predisposition between sexes. When stratified by intervention arm, the strength of predisposition between sexes was not significantly different within each arm.

## Discussion

This study provides evidence of moderate and persistent individual-level predisposition to *A. lumbricoides* infection over 7 years of follow-up in a longitudinal study in southern Ethiopia. Predisposition was observed consistently across intervention arms, age groups, and sexes. These findings are consistent with published longitudinal studies that have demonstrated aggregation of worm burdens and stable reinfection patterns in populations in areas of endemic infection.^7–12^

The large scale of the Geshiyaro project, both in terms of study duration and participant enrolment, allows for longitudinal monitoring of individuals’ infection status over many years of a control intervention programme. Here, data from the project’s sentinel sites have been analysed to assess the evidence for predisposition to *A. lumbricoides* infection.

Evidence for moderate individual-level predisposition to *A. lumbricoides* infection was observed across multiple years in the Gehsiyaro project. More individuals cleared infection in Arms 1 and 2 (cMDA) than in Arm 3 (sMDA), likely reflecting differences in treatment effectiveness. However, temporal correlations in infection status were consistent across all intervention arms, with stronger correlations between consecutive surveys and weaker correlations as the interval between surveys increased. Similarly, comparable levels of predisposition across all arms, as measured by Kendall’s W, indicate that heterogeneity in infection risk remains consistent across location and treatment strategies. This stability likely reflects persistent individual- or household-level factors, such as environmental exposure or host behaviour. However, as treatment frequency and individual compliance were not included in the analysis, their potential contribution to these patterns cannot be assessed.

Across all intervention arms, most woredas showed a significant concordance in infection status; however, the strength of predisposition varied. This spatial heterogeneity may reflect differences in environmental exposure, WaSH conditions, or treatment coverage between woredas. In some woredas Kendall’s W was not statistically significant, which may reflect low infection prevalence or limited variation in infection status across survey rounds, rather than the absence of true predisposition. In Arm 2, the strength of concordance increased with the proportion of infected individuals, suggesting that higher infection prevalence may partly amplify detectable predisposition. Differences in predisposition between woredas were generally modest, which likely explains why no differences were observed when results were aggregated at the intervention arm level.

Kebele-level analyses showed that predisposition was broadly consistent across sentinel sites within each intervention arm. Although higher infection prevalence was associated with higher W values when all sentinel sites were combined, this relationship was not observed when analyses were stratified by intervention arm. This suggests that the association was driven by differences between arms rather than by local prevalence. It is also possible that detecting differences in predisposition at the sentinel site level was limited by small sample sizes.

Age-stratified analyses showed consistently moderate and comparable levels of predisposition across all age groups. Differences in Kendall’s W were small and not explained by variation in infection prevalence or sample size, indicating that while age may influence exposure and infection intensity, individual-level predisposition persists across age groups. This is notable, as children are typically considered at higher risk of *A. lumbricoides* infection due to increased exposure.^19^ The lack of strong age-dependence suggests that while age may influence overall exposure, individual-level predisposition remains relatively stable across the lifespan, potentially reflecting persistent individual behaviour- or household-level factors.

Similarly, predisposition did not differ meaningfully by sex. Males and females had equivalent Kendall’s W values, and stratified analyses within intervention arms did not reveal sex-specific patterns of concordance. This suggests that sex-related differences in exposure or behaviour, if present, do not translate into systematic differences in long-term infection ranking.

The specific causes of predisposition to *A. lumbricoides* infection remain poorly understood and are probably multifactorial. The paucity of epidemiological studies investigating the key covariates influencing predisposition highlights a gap in understanding. Individual treatment histories were not incorporated into the present analysis and may contribute to observed patterns in persistent infection. Future studies should include longitudinal treatment histories to disentangle the effects of treatment non-compliance from intrinsic susceptibility to reinfection. In addition, incorporating behavioural and WaSH exposure data would help identify environmental and behavioural drivers of risk. Finally, molecular epidemiological approaches, such as parasite whole-genome sequencing and ‘who-infects-whom’ analyses, are needed to determine whether individuals with persistent infection contribute disproportionately to onward transmission within communities. A pilot study employing this approach has recently been published; however, larger longitudinal cohort studies are required.^20^

The findings presented here have implications for STH control and elimination strategies. Evidence for individual-level predisposition suggests that a small subset of individuals may contribute disproportionately to ongoing transmission. In transmission settings where STH elimination is being considered, identifying individuals, households, or communities who are susceptible to repeated reinfection could help prioritize them for targeted drug administration. This approach would be beneficial in settings where the continued donation of drugs by pharmaceutical companies is uncertain. A limitation of such an approach is that it would require diagnostic testing to identify predisposed individuals, whereas most current control programmes rely on mass treatment without prior diagnosis. However, if predisposition is stable over time, diagnostic testing may only need to be performed once rather than before each round of treatment.

While such a targeted approach could improve the sustainability of control programmes, it also raises ethical and social concerns. Classifying individuals as predisposed to reinfection could lead to compliance issues and potential stigma. This must be carefully considered in the development and implementation of targeted treatment strategies.

### Limitations

This study has several limitations. First, infection status was measured using Kato–Katz, which has reduced sensitivity in low-prevalence settings and may underestimate true prevalence.^20^ This underscores the potential value of molecular diagnostic methods, such as quantitative PCR, which are more sensitive in low-prevalence settings.^21,22^

Second, the longitudinal design resulted in some loss to follow-up, meaning that not all individuals had infection data for every sentinel site survey. This reduces the number of complete infection histories available for analysis and may limit the ability to detect consistent patterns of predisposition over time.

While this study interprets predisposition in the context of transmission persistence, it does not address the contribution of predisposed individuals to environmental contamination or onward transmission. It therefore cannot be concluded whether these individuals act as core transmitters within the population. As such, resolving this question will require molecular epidemiological studies, including parasite genome sequencing, to infer transmission patterns. However, independent of their role in sustaining transmission, individuals who are repeatedly infected may experience a disproportionate burden of chronic morbidity. Therefore, even in the absence of evidence for increased transmission, identifying such individuals remains important from a public health perspective.

## Conclusions

The findings derived from a subsample of the population in Wolayita provide evidence for individual-level predisposition to *A. lumbricoides* infection, consistent across districts, ages, and sexes, and persisting, despite repeated treatment rounds, over a 7-year period. Predisposition in endemic settings with low STH prevalence highlights the potential value of targeted interventions. As programmes move toward transmission elimination, detecting and interpreting predisposition becomes increasingly challenging, but remains important for identifying individuals or subgroups at persistent risk who sustain transmission within their communities. As drug donations reduce, targeted approaches may offer a more cost-effective and impactful alternative to blanket prophylactic MDA. Future research should focus on identifying those factors driving predisposition and on developing affordable, field-friendly diagnostics to support targeted control strategies.

## Supplementary Material

trag075_Supplemental_Files

## Data Availability

The data underlying this article will be shared upon reasonable request to the corresponding author.
