# Supplementary material for: Predisposition to Ascaris lumbricoides reinfection in the Geshiyaro project in southern Ethiopia
Source: Trans R Soc Trop Med Hyg. 2026 Jul 6;120(10):1068–77. doi: 10.1093/trstmh/trag075 (PMC13635670; doi:10.1093/trstmh/trag075)
Supplement: Supplementary file 1 [file trag075_supplemental_files.zip › Supplementary_table_1.docx]

| Activity | Project Year | n | Date |
| --- | --- | --- | --- |
| Sentinel site | 1 | 451 | 2019-01 |
| MDA | 1 | NA | 2019-02 |
| Sentinel site | 2 | 531 | 2019-10 |
| MDA | 2 | NA | 2019-11 |
| Sentinel site | 3 | 511 | 2020-10 |
| MDA | 3 | NA | 2020-10 |
| Sentinel site | 4 | 575 | 2021-10 |
| MDA | 4 | NA | 2021-10 |
| MDA | 4 | NA | 2022-05 |
| Sentinel site | 5 | 593 | 2022-10 |
| MDA | 5 | NA | 2022-11 |
| MDA | 5 | NA | 2023-06 |
| Sentinel site | 6 | 589 | 2023-12 |
| MDA | 6 | NA | 2024-01 |
| MDA | 6 | NA | 2024-08 |
| Sentinel site | 7 | 597 | 2024-12 |
| MDA | 7 | NA | 2025-02 |
| MDA | 7 | NA | 2025-10 |
