# Supplementary material for: Predisposition to Ascaris lumbricoides reinfection in the Geshiyaro project in southern Ethiopia
Source: Trans R Soc Trop Med Hyg. 2026 Jul 6;120(10):1068–77. doi: 10.1093/trstmh/trag075 (PMC13635670; doi:10.1093/trstmh/trag075)
Supplement: Supplementary file 1 [file trag075_supplemental_files.zip › Supplementary_table_2.docx]

| Arm | Activity | Project Year | n | Date |
| --- | --- | --- | --- | --- |
| 1 | Sentinel site | 2 | 1351 | 2020-02 |
| 2 | Sentinel site | 2 | 2131 | 2020-02 |
| 1 & 2 | MDA | 2 | NA | 2020-11 |
| 1 | Sentinel site | 3 | 1542 | 2021-10 |
| 2 | Sentinel site | 3 | 2061 | 2021-10 |
| 1 & 2 | MDA | 3 | NA | 2021-12 |
| 1 | Sentinel site | 4 | 1530 | 2022-05 |
| 2 | Sentinel site | 4 | 2069 | 2022-05 |
| 1 & 2 | MDA | 4 | NA | 2022-06 |
| 1 & 2 | MDA | 4 | NA | 2022-12 |
| 1 | Sentinel site | 5 | 1592 | 2023-06 |
| 2 | Sentinel site | 5 | 2129 | 2023-06 |
| 1 & 2 | MDA | 5 | NA | 2023-07 |
| 1 & 2 | MDA | 5 | NA | 2024-01 |
| 1 | Sentinel site | 6 | 1593 | 2024-07 |
| 2 | Sentinel site | 6 | 2175 | 2024-07 |
| 1 & 2 | MDA | 6 | NA | 2024-08 |
| 1 & 2 | MDA | 6 | NA | 2025-03 |
| 1 | Sentinel site | 7 | 1603 | 2025-07 |
| 2 | Sentinel site | 7 | 2165 | 2025-07 |
| 1 & 2 | MDA | 7 | NA | 2025-09 |
