# Supplementary material for: Predisposition to Ascaris lumbricoides reinfection in the Geshiyaro project in southern Ethiopia
Source: Trans R Soc Trop Med Hyg. 2026 Jul 6;120(10):1068–77. doi: 10.1093/trstmh/trag075 (PMC13635670; doi:10.1093/trstmh/trag075)
Supplement: Supplementary file 1 [file trag075_supplemental_files.zip › Supplementary_table_3.docx]

| Project year | n | Date |
| --- | --- | --- |
| 3 | 2093 | 2021-04 |
| 4 | 2177 | 2022-02 |
| 5 | 2191 | 2023-04 |
| 6 | 2231 | 2024-04 |
| 7 | 2236 | 2025-04 |
