# Supplementary material for: Predisposition to Ascaris lumbricoides reinfection in the Geshiyaro project in southern Ethiopia
Source: Trans R Soc Trop Med Hyg. 2026 Jul 6;120(10):1068–77. doi: 10.1093/trstmh/trag075 (PMC13635670; doi:10.1093/trstmh/trag075)
Supplement: Supplementary file 1 [file trag075_supplemental_files.zip › Supplementary_table_4.docx]

| Woreda comparison | Lower CI | Upper CI | Significance |
| --- | --- | --- | --- |
| Damot Sore vs Bolosso Sore | -0.15 | -0.04 | Yes |
| Damot Sore vs Bolosso Bombe | -0.13 | -0.04 | Yes |
| Damot Sore vs Damot Gale | -0.08 | -0.01 | Yes |
| Damot Sore vs Damot Pulassa | -0.12 | -0.01 | Yes |
| Bolosso Sore vs Bolosso Bombe | -0.04 | 0.06 | No |
| Bolosso Sore vs Damot Gale | <0.001 | 0.10 | Yes |
| Bolosso Sore vs Damot Pulassa | -0.04 | 0.09 | No |
| Bolosso Bombe vs Damot Gale | 0.01 | 0.08 | Yes |
| Bolosso Bombe vs Damot Pulassa | -0.04 | 0.07 | No |
| Damot Gale vs Damot Pulassa | -0.08 | 0.03 | No |
