# Supplementary material for: Predisposition to Ascaris lumbricoides reinfection in the Geshiyaro project in southern Ethiopia
Source: Trans R Soc Trop Med Hyg. 2026 Jul 6;120(10):1068–77. doi: 10.1093/trstmh/trag075 (PMC13635670; doi:10.1093/trstmh/trag075)
Supplement: Supplementary file 1 [file trag075_supplemental_files.zip › Supplementary_table_5.docx]

| Woreda comparison | Lower CI | Upper CI | Significance |
| --- | --- | --- | --- |
| Offa vs Damot Weydie | 0.02 | 0.13 | Yes |
| Offa vs Abela Abaya | 0.03 | 0.14 | Yes |
| Offa vs Kindo Didaye | -0.18 | -0.04 | Yes |
| Offa vs Sodo Zuria | 0.01 | 0.15 | Yes |
| Offa vs Diguna Fango | 0.01 | 0.12 | Yes |
| Offa vs Bodity | -0.01 | 0.13 | No |
| Damot Weydie vs Abela Abaya | -0.01 | 0.03 | No |
| Damot Weydie vs Kindo Didaye | -0.23 | -0.14 | Yes |
| Damot Weydie vs Sodo Zuria | -0.03 | 0.04 | No |
| Damot Weydie vs Diguna Fango | -0.03 | 0.01 | No |
| Damot Weydie vs Bodity | -0.04 | 0.02 | No |
| Abela Abaya vs Kindo Didaye | -0.24 | -0.15 | Yes |
| Abela Abaya vs Sodo Zuria | -0.04 | 0.03 | No |
| Abela Abaya vs Diguna Fango | -0.05 | -0.01 | Yes |
| Abela Abaya vs Bodity | -0.05 | 0.01 | No |
| Kindo Didaye vs Sodo Zuria | 0.13 | 0.24 | Yes |
| Kindo Didaye vs Diguna Fango | 0.12 | 0.22 | Yes |
| Kindo Didaye vs Bodity | 0.12 | 0.23 | Yes |
| Sodo Zuria vs Diguna Fango | -0.06 | 0.03 | No |
| Sodo Zuria vs Bodity | -0.06 | 0.03 | No |
| Diguna Fango vs Bodity | -0.03 | 0.03 | No |
