# Supplementary material for: Predisposition to Ascaris lumbricoides reinfection in the Geshiyaro project in southern Ethiopia
Source: Trans R Soc Trop Med Hyg. 2026 Jul 6;120(10):1068–77. doi: 10.1093/trstmh/trag075 (PMC13635670; doi:10.1093/trstmh/trag075)
Supplement: Supplementary file 1 [file trag075_supplemental_files.zip › Supplementary_table_6.docx]

| Woreda comparison | Lower CI | Upper CI | Significance |
| --- | --- | --- | --- |
| Wondo vs Wondo Genet | -0.02 | 0.10 | No |
| Wondo vs Tula Kifle Ketema | -0.01 | 0.11 | No |
| Wondo Genet vs Tula Kifle Ketema | -0.01 | 0.04 | No |
