# Supplementary material for: Predisposition to Ascaris lumbricoides reinfection in the Geshiyaro project in southern Ethiopia
Source: Trans R Soc Trop Med Hyg. 2026 Jul 6;120(10):1068–77. doi: 10.1093/trstmh/trag075 (PMC13635670; doi:10.1093/trstmh/trag075)
Supplement: Supplementary file 1 [file trag075_supplemental_files.zip › Supplementary_table_7.docx]

| Age group comparison | Lower CI | Upper CI | Significance |
| --- | --- | --- | --- |
| 36+ vs 21-35 | -0.01 | -0.02 | Yes |
| 36 + vs 15-20 | -0.15 | -0.06 | Yes |
| 36+ vs 5-14 | -0.11 | -0.04 | Yes |
| 36+ vs 0-4 | -0.07 | -0.01 | Yes |
| 21-35 vs 15-20 | -0.09 | -0.01 | Yes |
| 21-35 vs 5-14 | -0.05 | 0.02 | No |
| 21-35 vs 0-4 | -0.01 | 0.05 | No |
| 15-20 vs 5-14 | -0.01 | 0.07 | No |
| 15-20 vs 0-4 | 0.03 | 0.11 | Yes |
| 5-14 vs 0-4 | 0.01 | 0.07 | Yes |
